# Neuroanatomical location of brain metastases from solid tumours based on pathology: An analysis of 511 patients with a comparison to the provided clinical history

**DOI:** 10.1371/journal.pone.0294154

**Published:** 2023-11-09

**Authors:** Michael Bonert, Alison Berzins, Housne Begum, Jens Schittenhelm, Jian-Qiang Lu, Rosalyn A. Juergens, Anand Swaminath, Jean-Claude Cutz, Asghar H. Naqvi

**Affiliations:** 1 Anatomical Pathology, Pathology and Molecular Medicine, St. Joseph’s Healthcare Hamilton, McMaster University, Hamilton, Canada; 2 McMaster University, Hamilton, and Health Services Management, Toronto Metropolitan University, Toronto, Canada; 3 Department of Neuropathology, Institute of Pathology and Neuropathology, University Hospital of Tuebingen, Eberhard Karls University of Tuebingen, Tübingen, Germany; 4 Neuropathology, Pathology and Molecular Medicine, Hamilton Health Sciences, McMaster University, Hamilton, Canada; 5 Medical Oncology, Oncology, Hamilton Health Sciences, McMaster University, Hamilton, Canada; 6 Radiation Oncology, Oncology, Hamilton Health Sciences, McMaster University, Hamilton, Canada; University of Turin, S. Anna Hospital, ITALY

## Abstract

Brain metastases are a frequent occurrence in neuropathology practices. The literature on their neuroanatomical location is frequently derived from radiological analyses. This work examines brain metastases through the lens of pathology specimens. All brain surgical pathology reports for cases accessioned 2011–2020 were retrieved from a laboratory. Specimens were classified by neuroanatomical location, diagnosis and diagnostic category with a hierarchical free text string-matching algorithm (HFTSMA) and also subsequently audited. All reports classified as probable metastasis were reviewed by a pathologist. The provided history was compared to the final categorization by a pathologist. The cohort had 4,625 cases. The HFTSMA identified 854 cases (including metastases from a definite primary, metastases from primary not known and improperly classified cases). 514/854 cases had one definite primary site per algorithm and on report review 538/854 cases were confirmed as such. The 538 cases originated from 511 patients. Primaries from breast, gynecologic tract, and gastrointestinal tract not otherwise specified were most frequently found in the cerebellum. Kidney metastases were most frequently found in the occipital lobe. Lung, metastatic melanoma and colorectal primaries were most commonly found in the frontal lobe. The provided clinical history predicted the primary in 206 cases (40.3%), was discordant in 17 cases (3.3%) and non-contributory in 280 cases (54.8%). The observed distribution of the metastatic tumours in the brain is dependent on the primary site. In the majority (54.8%) of cases, the provided clinical history was non-contributory; this suggests surgeon-pathologist communication may have the potential for optimization.

## Introduction

Brain metastases (BM) represent approximately 50% of brain tumours in adult patients and occur in up to 10–30% of cancer patients during follow-up [1]. The median interval between diagnosing primary tumour and brain metastases is 8.5 months [2]. However, in approximately 20% of the cases, BM are the first symptom of advanced cancer [3]. The pathologist’s task in these specimens is (i) diagnose the BM, (ii) pathologically type it/determine the primary site (if this is possible) and (iii) perform ancillary testing, i.e. biomarkers, required for patient-centred oncology treatment recommendations.

The location of brain metastases is known to depend on the primary site. Usually they spread hematogenously to the brain and/or spinal cord. Approximately 80% of BM are in the supratentorial brain parenchyma and predominantly located in between the white and grey matter junction and in anatomic watershed areas [4].

Several large epidemiological studies have been done on brain metastases. Evaluation of the Surveillance, Epidemiology, and End Results (SEER) database showed the highest age-standardized BM incidence rates for lung cancer, melanomas and breast cancer in a cohort of 66,655 patients [5]. However, screening for BM is not employed across all malignancies and thus asymptomatic brain metastases are often missed and diagnosis of BM in such registries in most studies is assumed on primary histology [6]. A retrospective review of 148 autopsy cases showed that (metastatic) malignant melanoma BM were associated with frontal and temporal lobes, breast carcinoma with cerebellum and lung cancer with the occipital lobe and cerebellum [7].

Studies employing neuropathological records of BM surgeries are limited. A neuropathological analysis of 459 cancer of unknown primary (CUP) tumours of the Vienna Brain Metastasis registry showed, that in 81.5% cases, an extracranial primary tumour, most commonly lung cancer, could be identified within 3 months [3]. *Capper et al*. collected 885 BM cases between 1990 and 2011 from the archives of a single institution for BRAF V600E mutation testing [8]. In that study, 40% of the samples originated from respiratory tract, 25% from urogenital/reproductive tract, 10% from gastrointestinal cancers and 8% were from the skin. Schroeder *et al*. examined 369 patients with an average of 3 BM per patients and found that the majority of their cases were located in the frontal lobes (31%) and in the cerebellum (24%) [9]. Pulmonary and gastrointestinal BM appeared more often in infratentorial regions, while skin BM were almost always restricted to a supratentorial location, again highlighting a spatial distribution dependent on primary site.

A metanalysis by Cardinal *et al*. [10] included 13 studies with radiologic BM identification and found that breast, lung, and colorectal cancer metastasized frequently into the cerebellum, with notable differences based on subtype and receptor expression.

BM distribution data is frequently derived from the radiologic distribution and is often focused on a primary diagnosis (eg. breast cancer cohort); it is expected to differ from (surgical and autopsy) neuropathological cohorts–which are driven by symptom management and/or diagnostic considerations (eg. What is the unknown primary? What treatment options may be available based on the tumour’s pathologic characteristics?). The current study aims to identify and type BM by employing a diagnostic algorithm to evaluate pathology reports retrospectively.

## Materials and methods

### Ethics statement

Ethics approval (Hamilton Integrated Research Ethics Board (HiREB) # 14453-C) was obtained to retrieve reports for all in house surgical pathology accessioned January 1, 2011 to December 31, 2020 at a regional laboratory, such that all in-house brain pathology cases could be retrieved for a retrospective analysis. The regional laboratory serves a neurosurgery team with a catchment of approximately 2.5 million people. Patient consent was not required by the ethics board, due to the study design. After data extraction, the dataset was anonymized by removing all patient identifiers.

All in-house brain pathology cases accessioned January 1, 2011 to December 31, 2020 were retrieved for analysis on May 12, 2022. The cases were classified by neuroanatomical location, diagnosis and diagnostic category with a hierarchical free text string-matching algorithm (HFTSMA). The diagnostic dictionary, diagnostic hierarchy and location dictionary are provided in the Appendix A in S2 File.

The project builds on work previously performed by our group dealing with lung core biopsies, [11] and on knowledge acquired parsing free text pathology reports [12,13].

Extracted diagnoses were grouped into six mutually exclusive groupings (primary brain tumour, metastasis, other brain lesion, miscellaneous lesions, brain cardiovascular disease, unclassified). The mutually exclusive groups definition is provided in the Appendix B in S2 File. The HFTSMA further subdivided the possible metastases group into three subgroups (Subgroup 1: cases with zero identified possible primary sites; Subgroup 2: cases with one identified possible primary site; Subgroup 3: cases with two or more identified possible primary sites).

All reports classified as possible metastasis by HFTSMA were reviewed by a pathologist. The confidence in the primary site was scored zero to three, where ‘score 0’ is ‘unknown primary/differential diagnosis is given’, ‘score 1’ is ‘possible primary X/considerable uncertainty exists in the primary site’, ‘score 2’ is ‘primary is X’ and ‘score 3’ is ‘primary X is proven’. Only cases that were ‘score 2’ or ‘score 3’ (by report review) were included in the analysis. In patients with multiple specimens with metastatic disease, the first specimen was retained and the remaining specimens purged from the data set. In the selected study set, all the neuroanatomical locations were reviewed by a pathologist to ensure accuracy.

**Fig 1:**
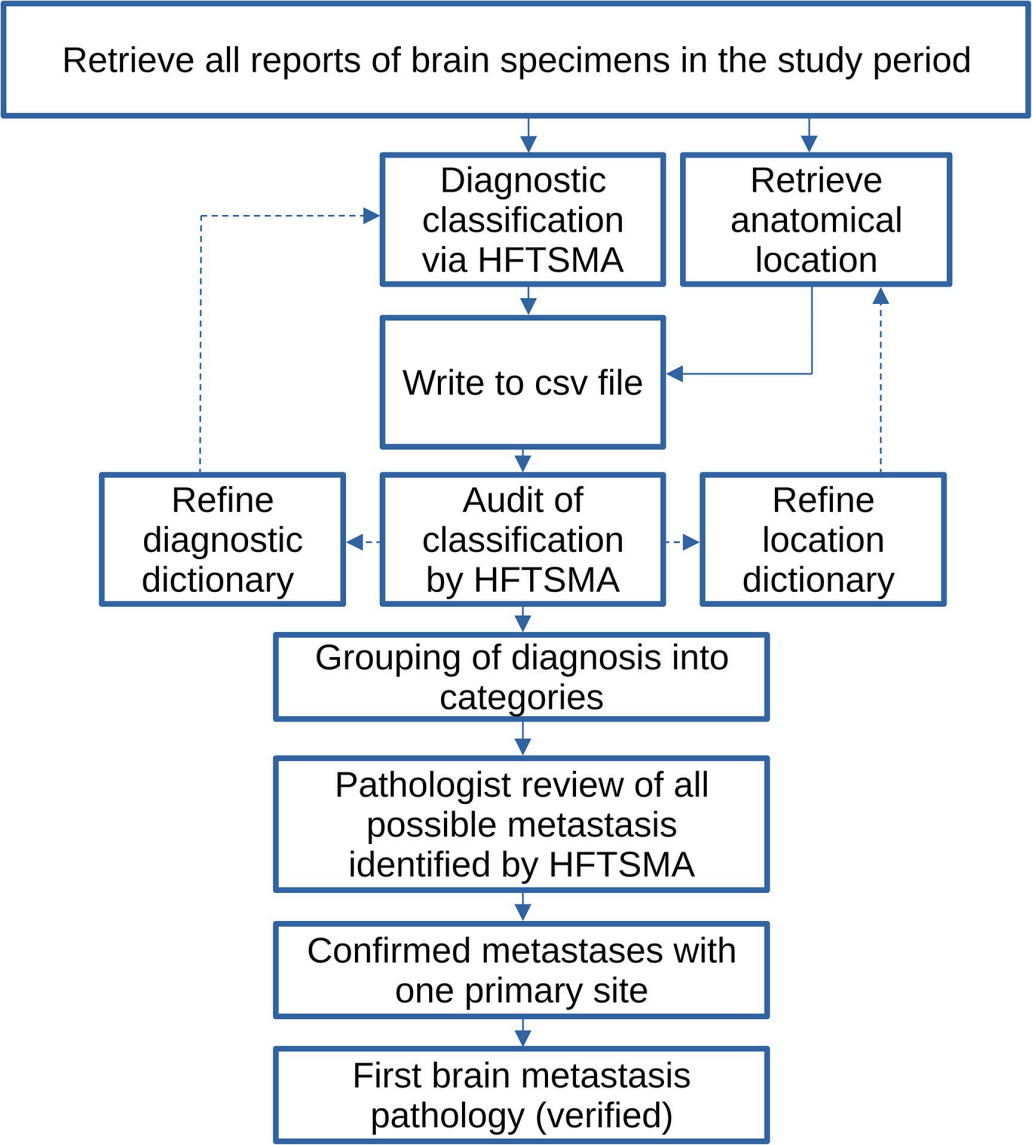
The study methods in a flowchart. HFTSMA = hierarchical free text string-matching algorithm; csv = formatted text output (tab or comma separated).

The methods are summarized in a flowchart—Fig 1.

## Results

The study had 4,625 brain pathology cases. The HFTSMA could classify 4,556 of 4,625 cases diagnostically and 4,577 of 4,625 were classified by neuroanatomical location. The classification codes went through approximately 30 iterations.

The unaudited result of the HFTSMA (after iteration) by mutually exclusive grouping were: primary brain tumour 2,613, metastasis 854, other brain lesion 264, miscellaneous lesions 87, brain cardiovascular disease 738, unclassified 69.

The HFTSMA categorization accuracy was 96% (192/200) in randomly selected cases and 78% (156/200) for the primary site in metastases; this prompted further human reviews of the reports. Only reviewed reports were included in the analysis. 514/854 of the metastases had one primary site and on review 538/854 cases were confirmed as such.

The HFTSMA further subdivided the 854 possible metastases into three subgroups. Subgroup 1 (primary site not identified by algorithm) had 182 cases; 26 were scored 2 or 3. Subgroup 2 (one primary site identified by algorithm) had 523 cases; 474 were scored 2 or 3 on final review. The cases in subgroup 2 were reviewed on two different occasions. In the first review of subgroup 2 cases were classified ‘include’ or ‘exclude’. The in second review approximately 20 additional cases were excluded; most of these were ‘score 1’. Subgroup 3 (more than one primary site identified by algorithm) had 149 cases; 38 were scored 2 or 3. In total, 538 cases were scored 2 or 3; these 538 cases were from 511 patients. The location classification was correct in 94% (480/511) of verified cases. More details on the case classification are found in S1 File (Cases Scoring by Subgroup).

### Patient with multiple specimens

Thirty patients had more than one brain pathology case with metastatic disease in the time period; one had four cases and 29 had two cases. In 26 of 29 patients with two cases the primary tumour site matched, e.g. melanoma on first case and melanoma on the second case. In the remaining three patients, the primaries likely matched. One of the 29 patients had small cell carcinoma followed by non-small cell carcinoma of the lung; however, immunostains were not done on the second case. In 20 of 29 patients with two specimens the neuroanatomical site of the first and subsequent specimen were the same. The patient with four brain cases had the first classified as ‘colorectal’; subsequent pathology was classified ‘GI NOS’, ‘colorectal’ and ‘adenocarcinoma not further specified [in the report]—but had a reference to the prior pathology’.

### First brain pathology with metastatic disease

The remaining analysis is focused on each patient’s first brain (metastatic disease) pathology specimen in the time frame. In the thirty patients that had multiple specimens with metastatic disease, all subsequent specimens were excluded.

The number of patients and lesions by neuroanatomical location and primary site is shown in Table 1 (Raw Data).

**Table 1:** Patients by primary site and Lesions by primary site and location.

Primary Site	Patients	Lesions	Frontal	Parietal	Temporal	Occipital	Cerebellum	Unk./Oth.
**Lung**	234	255	88	48	20	35	61	3
**Melanoma**	87	95	43	10	22	7	11	2
**Breast**	65	76	13	17	8	9	28	1
**Colorectal**	40	46	15	6	7	3	14	1
**Kidney**	20	20	5	1	1	7	5	1
**GI NOS**	17	20	3	4	0	5	8	0
**Gynecologic**	16	17	5	3	1	3	5	0
**Urothelial**	12	12	2	2	3	2	3	0
**Other Tumours**	20	21	8	2	2	2	5	2
**Sum**	511	562	182	93	64	73	140	10

Other Tumours = prostate, thyroid, head & neck cancer, germ cell tumour, sarcoma; NOS = not otherwise specified; Unk./Oth. = unknown or other location.

The neuroanatomical location could be determined in 507 of 511 patients; in four patients the location of the lesion was unknown. Lung (234 patients; 45.8%) was the most common primary site followed by melanoma (87 patients; 17.0%), breast (65 patients; 12.7%), colorectal (40 patients; 7.8%), renal (20 patients; 3.9%), GI NOS (17 patients; 3.3%), gynaecologic (16 patients; 3.1%), urothelial (12 patients; 2.3%), and other tumours (20 patients; 3.9%). (Table 1) The frontal lobe was the most commonly neuroanatomical site (182 specimens), followed by the cerebellum (140 specimens).

The 20 “other tumours” consisted of 4 prostate carcinomas, 4 sarcomas, 2 head and neck carcinomas, 6 metastatic germ cell tumours (5/6 testicular, 1/6 not specified/unknown) and 4 thyroid carcinomas.

**Fig 2:**
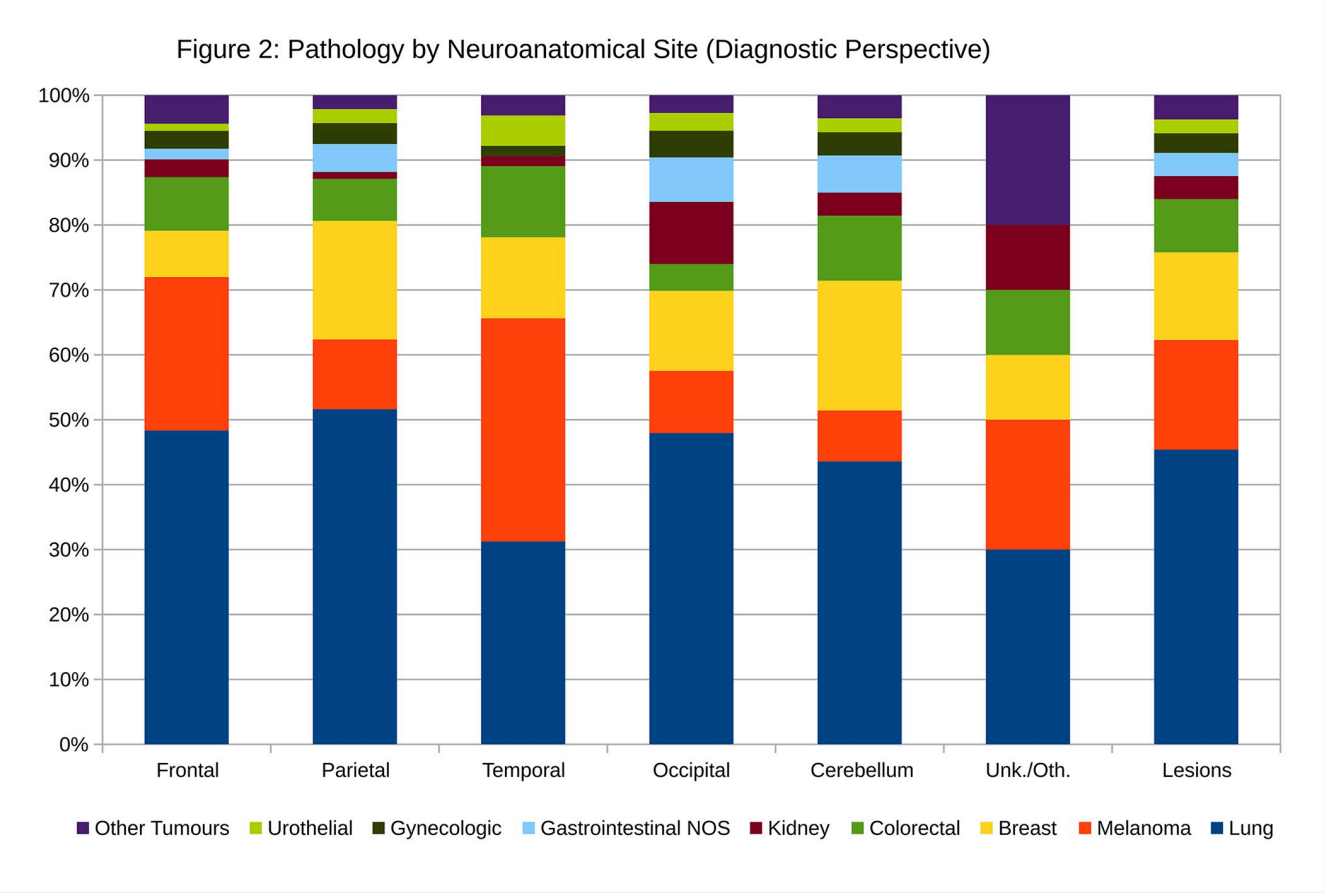
The brain metastases by neuroanatomical site. This figure is applicable in the diagnostic context; if the neuroanatomical site is known, it helps answer the question “How likely is a metastasis from a primary tumour that arose from ‘X’?”.

The pathology by neuroanatomical site is given in Table 2 (Cases by Neuroanatomical Location) and Fig 2. Lung was the most common primary in all (known) sites except the temporal lobe.

**Table 2:** Pathology by neuroanatomical site (Diagnostic Perspective).

Primary Site	Frontal	Parietal	Temporal	Occipital	Cerebellum	Unk./Oth.	Lesions
**Lung**	48.4%	51.6%	31.3%	47.9%	43.6%	30.0%	45.4%
**Melanoma**	23.6%	10.8%	34.4%	9.6%	7.9%	20.0%	16.9%
**Breast**	7.1%	18.3%	12.5%	12.3%	20.0%	10.0%	13.5%
**Colorectal**	8.2%	6.5%	10.9%	4.1%	10.0%	10.0%	8.2%
**Kidney**	2.7%	1.1%	1.6%	9.6%	3.6%	10.0%	3.6%
**GI NOS**	1.6%	4.3%	0.0%	6.8%	5.7%	0.0%	3.6%
**Gynecologic**	2.7%	3.2%	1.6%	4.1%	3.6%	0.0%	3.0%
**Urothelial**	1.1%	2.2%	4.7%	2.7%	2.1%	0.0%	2.1%
**Other Tumours**	4.4%	2.2%	3.1%	2.7%	3.6%	20.0%	3.7%
**Sum**	100.0%	100.0%	100.0%	100.0%	100.0%	100.0%	100.0%
**Lesions**	182	93	64	73	140	10	562

Other Tumours = prostate, thyroid, head & neck cancer, germ cell tumour, sarcoma; NOS = not otherwise specified; Unk./Oth. = unknown or other location.

**Fig 3:**
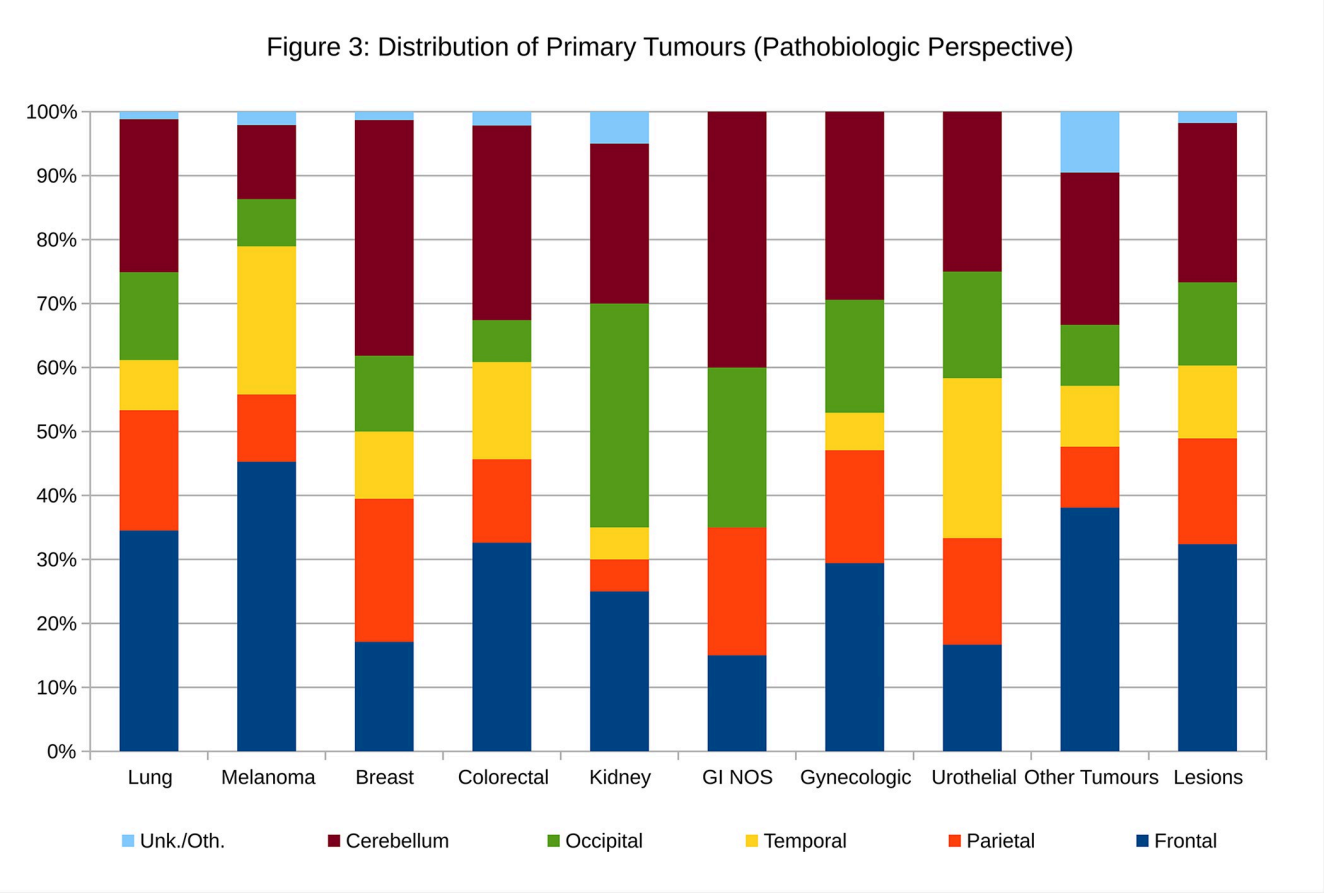
The brain metastases by primary site. This figure is applicable in the pathobiologic context; if the primary site is known, it helps answer the question “How likely is a metastasis to the neuroanatomical site ‘Y’?”.

The neuroanatomical distribution of the metastatic lesions by primary site is shown in Table 3 (Cases by Primary Site) and Fig 3.

**Table 3:** Distribution of metastatic tumours by primary site (Pathobiologic Perspective).

Primary Site	Lesions	Frontal	Parietal	Temporal	Occipital	Cerebellum	Unk./Oth.	Sum
Lung	255	34.5%	18.8%	7.8%	13.7%	23.9%	1.2%	100.0%
Melanoma	95	45.3%	10.5%	23.2%	7.4%	11.6%	2.1%	100.0%
Breast	76	17.1%	22.4%	10.5%	11.8%	36.8%	1.3%	100.0%
Colorectal	46	32.6%	13.0%	15.2%	6.5%	30.4%	2.2%	100.0%
Kidney	20	25.0%	5.0%	5.0%	35.0%	25.0%	5.0%	100.0%
GI NOS	20	15.0%	20.0%	0.0%	25.0%	40.0%	0.0%	100.0%
Gynecologic	17	29.4%	17.6%	5.9%	17.6%	29.4%	0.0%	100.0%
Urothelial	12	16.7%	16.7%	25.0%	16.7%	25.0%	0.0%	100.0%
Other Tumours	21	38.1%	9.5%	9.5%	9.5%	23.8%	9.5%	100.0%

Other Tumours = prostate, thyroid, head & neck cancer, germ cell tumour, sarcoma; NOS = not otherwise specified; Unk./Oth. = unknown or other location.

Primaries from breast, gynecologic tract (endometrium, ovary, cervix) and gastrointestinal tract not otherwise specified most frequently were found in the cerebellum. Kidney metastases were most frequently found in the occipital lobe. Lung, metastatic melanoma and colorectal primaries were most commonly found in the frontal lobe specimens.

In 98 patients (19%) the location codes overlapped/multiple locations were given. The overlap resulted from cases that included several keywords, e.g. ‘frontal’ and ‘temporal’ or multiple parts. The overlap is shown in Table 4 (Neuroanatomical Site Overlap).

**Table 4:** Neuroanatomical site overlap.

Site	Frontal	Parietal	Temporal	Occipital	Cerebellum	Other	Unknown	Misc.
**Frontal**	182	11	10	0	1	6	0	-
**Parietal**	11	93	10	17	0	3	0	-
**Temporal**	10	10	64	0	0	3	0	-
**Occipital**	0	17	0	73	3	4	0	-
**Cerebellum**	1	0	0	3	140	27	0	-
**Other**	6	3	3	4	27	47	0	-
**Unknown**	0	0	0	0	0	0	4	-
**Sum**	210	134	87	97	171	90	4	511
**Diagonal**	182	93	64	73	140	47	4	-
**Off-diagonal**	28	41	23	24	31	43	0	-
**Pure**	154	52	41	49	109	4	4	413
**Mixed**	-	-	-	-	-	-	-	98

### Provided clinical history

The provided clinical history predicted the primary in 206 cases (40.3%), was discordant in 17 cases (3.3%) and non-contributory in 280 cases (54.8%). Examples of “non-contributory” histories are: “Seizure. Left occipital tumour”, “Left supratentorial mass” and “Headaches with nausea/vomiting. Cerebellar tumour.” Examples of “discordant” histories are: “Personality changes. Metastatic breast cancer. Right frontal tumour.” where the diagnosis was ‘lung primary’ and “History of colon cancer—bowel resection.” where the diagnosis was ‘lung primary’. If the provided history had more than one possibility (e.g. “history of prostate cancer and melanoma”) and the diagnosis was one of those (e.g. “melanoma”) the case was classified as “concordant differential diagnosis”. One patient had no provided history at all; instead a placeholder was present (“Not available on requisition”). A summary of the findings is show in Table 5 (Clinical History).

**Table 5:** Clinical history.

Category	Count	Percent
**Discordant**	17	3.3%
**Not Relevant/Not Useful**	280	54.8%
**Concordant**	206	40.3%
**Concordant DDx**	8	1.6%
**Sum**	511	100.0%

DDx = differential diagnosis.

## Discussion

The project successfully extracted information from free text pathology reports using a hierarchical free text string-matching algorithm. As the accuracy was not deemed sufficiently high, all included reports were reviewed. It was apparent that some primary site classifications have a certain degree of subjectivity; however, we are confident that, in aggregate the trends within the data are robust. In patients with multiple brain metastasis specimens, later pathology was excluded—as it was likely due to the same underlying pathology and represents a different time point in the disease. Also, we did not want to ‘double count’ individuals.

Primaries from breast, gynecologic tract, and gastrointestinal tract not otherwise specified were most frequently found in the cerebellum. Kidney metastases were most frequently found in the occipital lobe. Lung, metastatic melanoma and colorectal primaries were most commonly found in the frontal lobe. The aggregated surgical pathology results appear to be useful to understand patterns of spread and largely matches the spread pattern observed radiologically. Data of this type may be useful to better understand the biology of metastatic disease.

This cohort represents a limited perspective into metastatic brain cancer, as many metastatic brain lesions are not biopsied or excised. Uncommon metastatic lesions (e.g. metastatic gestational trophoblastic neoplasia [14] may not be well represented. Asymptomatic cases may be missed because not all entities undergo regular brain imaging. Brain biopsies are typically taken from non-critical sites. Larger lesions and easy to access neuroanatomical sites (e.g. frontal lobe [15]) are favoured biopsy targets. It is not surprising that the frontal lobe was the most targeted neuroanatomical site in the cohort.

Lesions that are biopsied are usually due to one of the following scenarios: (i) radiologically suspected metastasis and unknown primary, (ii) prove that a candidate primary lesion (as may be ascertained via cross-sectional imaging, clinical exam or history) is the source of the lesion in the brain, (iii) prove a prior known cancer has recurred in the brain/determine which cancer has spread to brain if the patient had several primaries and (iv) removing a BM to reduce neurological symptoms (e.g. removal of cerebellar lesion to reduce ataxia). These scenarios represent a specific time point in the progression of a cancer and are also reflective of the biology to a certain degree.

A limitation of the study is that it is focused exclusively on metastases where the primary site could be determined from the pathology report. Brain cancer of unknown primary (CUP) is a significant subset of brain metastatic disease (possibly ~130–210) patients in the local brain biopsy/resection cohort); however, it is one that is likely a heterogenous group and one that is quite different than the group examined. In up to 45% of CUP cases, a solitary BM is the first manifestation of systemic cancer [16].

Ancillary testing for some specific tumours (e.g. SMARCA4-deficient (lung) adenocarcinoma–which is typically TTF-1 negative) is not available or not done routinely on brain metastatic specimens (e.g. lung biomarker panel); this limits the ability of the pathologist to classify some tumours. Even in the context of state-of-the-art testing: a relevant clinical history is essential for a number of primary tumours (e.g. squamous cell carcinoma, foregut derived (gastrointestinal tract) carcinomas, urothelial carcinoma, poorly differentiated tumours); in some cases, tumours can only be classified with a high degree of certainty with a thorough work-up—that includes imaging, past medical history, exposure history and family history.

### Provided clinical history

Clinical history was provided for the vast majority of patients and this exceeded expectations, based on a prior study [17]. However, the lack of a provided concordant history (54.8%) suggests communication between surgery and pathology may benefit from optimization. A relevant clinical history may speed turn-around time, minimize unnecessary ancillary test use, and ensure appropriate biomarker testing is done.

In the current practice paradigm, information is frequently siloed within departments and complicated by the use of different electronic systems. For example, pathologists and radiologists often use different electronic data repositories; the result is that relevant extractable information (in imaging reports) is typically not transferred (automatically) to pathology.

A general trend in pathology is: synoptic reporting; this has been found to increase completeness [18] and has been mandated for cancer cases in Ontario, Canada [19] and mandated by law in California, United States [20,21].

We propose that neurosurgeons should (as a standard of communication), in the context of a brain tumour biopsy, always provide (via a synoptic type requisition) three key elements to the neuropathologist: (i) history of prior malignancy (proven by pathology), (ii) suspected primary site (iii) primary objective (diagnosis (biopsy) versus symptom management (excision) versus both (excisional biopsy)). Well designed and properly implemented medical information systems could help facilitate the information flow, improve communication and quality.

### Neuroanatomical location

Cases with multiple neuroanatomical sites complicated the analysis. As the number of cases with multiple location codes (98/511) were a minority of cases, a preliminary (simplified) analysis with one neuroanatomical site per patient reproduced all the same trends.

Lesions with multiple neuroanatomical sites likely represent a slightly different disease (singular brain metastasis versus multiple brain metastases).

Cases with multiple parts sampling the same neuroanatomical location, e.g. ‘frontal’ may be submitted in two specimens or parts (e.g. Part A and Part B). The parts may represent two samples from the same lesion (most likely) or two samples from different lesions in the same neuroanatomical site (e.g. ‘frontal lesion #1’ and ‘frontal lesion #2’). Cases that sample multiple adjacent lobes (e.g. ‘fronto-parietal’) may be one large lesion or two different lesions in different lobes. It is not possible to definitely determine the scenario from the pathology report, as the information is not contained within.

## Conclusion

This is one of the largest cross-sectional surgical pathology studies to examine metastatic brain tumours by neuroanatomical location to date. It represents a unique window into the biology of metastatic brain cancer, as pathologists see a unique subset of brain metastases on biopsy. The distribution of the metastatic tumours in the brain mirror findings in other studies and largely matches patterns observed radiologically. In the majority of brain metastases, the provided clinical history was non-contributory; this suggests surgeon-pathologist communication may have the potential for optimization. Future work could correlate the pathologic findings and/or biomarker typing with radiologic findings to obtain a more complete picture.

## Supporting information

S1 File (ODS)

S2 File (ODT)
