# Supplementary material for: Neuroanatomical location of brain metastases from solid tumours based on pathology: An analysis of 511 patients with a comparison to the provided clinical history
Source: PLoS One. 2023 Nov 9;18(11):e0294154. doi: 10.1371/journal.pone.0294154 (PMC10635465; doi:10.1371/journal.pone.0294154)
Supplement: Supplementary file 1 [file pone.0294154.s002.odt]

# Appendices

### Appendix A

Diagnostic Codes and Text

| Diagnostic code | Text |
| --- | --- |
| ndx01 | glioblastoma |
| ndx02 | anaplastic astrocytoma |
| ndx03 | astrocytoma |
| ndx04 | anaplastic oligodendroglioma |
| ndx05 | oligodendroglioma |
| ndx06 | Anaplastic ganglioglioma |
| ndx07 | Medulloblastoma |
| ndx08 | ganglioglioma |
| ndx09 | Mixed glioneuronal tumour |
| ndx09 | Mixed glioneuronal tumor |
| ndx10 | gliosarcoma |
| ndx11 | Glioneuronal tumour with neuropil islands |
| ndx11 | Glioneuronal tumor with neuropil islands |
| ndx12 | Glioneurocytoma |
| ndx13 | Central neurocytoma |
| ndx14 | astrocytic tumour |
| ndx15 | Hemangiopericytoma |
| ndx16 | Anaplastic hemangiopericytoma |
| ndx17 | anaplastic gangliocytic tumour |
| ndx17 | anaplastic gangliocytic tumor |
| ndx18 | Dysplastic gangliocytoma |
| ndx18 | dysplastic cerebellar gangliocytoma |
| ndx18 | Lhermitte-Duclos disease |
| ndx19 | Pleomorphic xanthoastrocytoma |
| ndx20 | Xanthoastrocytoma |
| ndx22 | oligoastrocytoma |
| ndx23 | dysembryoplastic neuroepithelial tumour |
| ndx23 | dysembryoplastic neuroepithelial tumor |
| ndx24 | Subependymoma |
| ndx25 | Ependymoma |
| ndx26 | pilocytic astrocytoma |
| ndx27 | liponeurocytoma |
| ndx28 | Craniopharyngioma |
| ndx29 | gliosis |
| ndx30 | Glioma |
| ndx31 | Pineoblastoma |
| ndx32 | Pineal parenchymal tumor |
| ndx32 | Pineal parenchymal tumour |
| ndx33 | Pineocytoma |
| ndx34 | Germinoma |
| ndx35 | Dysgerminoma |
| ndx36 | Primitive neuroectodermal tumour |
| ndx36 | Primitive neuroectodermal tumor |
| ndx37 | cerebral neuroblastoma |
| ndx38 | Low grade glioneurocytic tumour |
| ndx38 | Low grade glioneurocytic tumor |
| ndx39 | pituicytoma |
| ndx40 | Gangliocytoma |
| ndx41 | hemangioblastoma |
| ndx42 | rosetting glioneuronal tumour |
| ndx42 | rosetting glioneuronal tumor |
| ndx43 | Multinodular and vacuolating neuronal tumour with associated focal cortical dysplasia |
| ndx43 | Multinodular and vacuolating neuronal tumor with associated focal cortical dysplasia |
| ndx44 | glioneuronal papillary tumour |
| ndx44 | glioneuronal papillary tumor |
| ndx46 | Choroid plexus papilloma |
| ndx47 | Chordoma |
| ndx48 | Cavernous hemangioma |
| ndx48 | cavernous angioma |
| ndx49 | Inverted papilloma |
| ndx50 | aneurysmal bone cyst |
| ndx52 | neuroepithelial cyst |
| ndx53 | encephalocele |
| ndx54 | Rathke cleft cyst |
| ndx54 | Rathke's cleft cyst |
| ndx54 | Rathke's type cyst |
| ndx54 | Rathke's cyst |
| ndx54 | cyst (consistent with Rathke type) |
| ndx54 | Rathke cyst |
| ndx55 | colloid cyst |
| ndx55 | Neuroepithelial (colloid type) cyst |
| ndx56 | Dermoid cyst |
| ndx57 | Epidermoid cyst |
| ndx58 | abscess |
| ndx59 | subdural empyema |
| ndx60 | cerebral toxoplasmosis |
| ndx61 | Aspergillus |
| ndx65 | primary angitis |
| ndx66 | CAPNON |
| ndx66 | calcifying pseudoneoplasm of the neuraxis |
| ndx67 | sarcoid |
| ndx68 | Lymphocytic hypophysitis |
| ndx69 | Giant cell tumour of bone |
| ndx70 | meningioma |
| ndx70 | meninigioma |
| ndx70 | Menginioma |
| ndx71 | schwannoma |
| ndx72 | Pituitary adenoma |
| ndx73 | Pituitary microadenoma |
| ndx80 | Progressive multifocal leukencephalopathy |
| ndx80 | progressive multifocal leukoencephalopathy |
| ndx81 | Arteriovenous malformation |
| ndx82 | vascular malformation |
| ndx83 | Spongiform encephalopathy |
| ndx83 | Creutzfeldt-Jakob disease |
| ndx84 | demyelinating plaque |
| ndx90 | Diffuse B-cell large cell lymphoma |
| ndx90 | Diffuse large B-cell lymphoma |
| ndx90 | Diffuse large cell high grade lymphoma, most likely B-cell type |
| ndx90 | Diffuse B-cell lymphoma, large cell |
| ndx90 | Diffuse Large B cell lymphoma |
| ndx130 | Atherosclerotic plaque |
| ndx130 | atheromatous plaque |
| ndx141 | subdural hematoma |
| ndx141 | subdural hemorrhage |
| ndx142 | intracerebral hematoma |
| ndx142 | Intracerebral hemorrhage |
| ndx143 | cerebellar hemorrhage |
| ndx144 | Acute cerebral hemorrhage |
| ndx145 | Acute hemorrhage |
| ndx146 | epidural hematoma |
| ndx146 | epidural hemorrhage |
| ndx147 | Hemorrhage, recent |
| ndx148 | subarachnoid hemorrhage |
| ndx148 | subarachnoid hematoma |
| ndx149 | Brain with hemorrhagic infarction |
| ndx150 | subdural membrane |
| ndx175 | Platelet fibrin thrombus, mixed type |
| ndx175 | Red cell rich, mixed thrombus |
| ndx176 | Platelet fibrin thrombus, red cell rich |
| ndx176 | Platelet fibrin thrombi admixed with red blood cells |
| ndx176 | thrombus, red blood cell rich |
| ndx177 | Red blood cell and fibrin aggregates, consistent with thrombosis |
| ndx177 | Red blood cell and fibrin aggregates, compatible with thrombosis |
| ndx177 | Fibrin and red blood cell aggregates, compatible with thrombosis |
| ndx177 | Fibrin and red blood cell aggregates, consistent with thrombosis |
| ndx178 | thrombus |
| ndx178 | thrombosis |
| ndx178 | clot |
| ndx179 | hematoma |
| ndx179 | hemorrhage |
| ndx179 | hemorrhagic tissue |
| ndx180 | Normal anterior pituitary gland |
| ndx180 | Normal pituitary |
| ndx181 | Normal cortex |
| ndx182 | Normal brain |
| ndx190 | no features of a specific infectious process or neoplastic process |
| ndx190 | no features diagnostic of neoplasm or primary inflammation |
| dx02 | adenocarcinoma |
| dx03 | squamous carcinoma |
| dx03 | squamous cell carcinoma |
| dx04 | small cell carcinoma |
| dx04 | small cell type carcinoma |
| dx04 | small cell neuroendocrine carcinoma |
| dx05 | non-small cell carcinoma |
| dx05 | non small cell carcinoma |
| dx05 | nonsmall cell carcinoma |
| dx05 | non-small cell type of carcinoma |
| dx06 | large cell neuroendocrine carcinoma |
| dx07 | atypical carcinoid |
| dx08 | typical carcinoid |
| dx08 | carcinoid |
| dx08 | neuroendocrine tumor |
| dx09 | Atypical mesothelial hyperplasia |
| dx10 | mesothelioma |
| dx15 | Atypical glandular proliferation |
| dx15 | Atypical adenomatous hyperplasia |
| dx15 | atypical alveolar hyperplasia |
| dx15 | atypical alveolar cells |
| dx15 | atypical bronchioalveolar proliferation |
| dx20 | malignant |
| dx20 | malignant tumor |
| dx20 | malignant tumour |
| dx20 | malignant neoplasm |
| dx20 | consistent with malignancy |
| dx20 | Poorly differentiated neoplasm |
| dx20 | Poorly differentiated malignancy |
| dx20 | Malignant epithelial neoplasm |
| dx20 | MALIGNANT GLANDS SEEN |
| dx20 | Malignant glandular neoplasm |
| dx20 | Myxoid neoplasm |
| dx20 | Mucinous neoplasm |
| dx20 | Invasive Epitheloid neoplasm |
| dx20 | Positive for epithelial neoplasm |
| dx21 | LYMPHOPROLIFERATIVE DISORDER |
| dx21 | lymphoproliferative process |
| dx21 | chronic lymphocytic leukemia |
| dx21 | monotonous lymphoid infiltrate |
| dx21 | Atypical lymphoid aggregate |
| dx21 | Atypical lymphoid infiltrate |
| dx21 | suggestive of lymphocytic proliferation |
| dx21 | suspicious for lymphoplasmacytic proliferative disorder |
| dx21 | Dense lymphocytic infiltrative proliferation |
| dx22 | lymphoma |
| dx23 | sarcoma |
| dx23 | sarcoma, |
| dx25 | Soft tissue neoplasm |
| dx25 | spindle cell lesion |
| dx25 | Spindle cell neoplasm |
| dx27 | suspect for neoplasm |
| dx27 | atypical cells identified |
| dx27 | atypical cells present |
| dx27 | Few atypical cells |
| dx27 | atypical but degenerated cells |
| dx27 | atypical, but degenerated cells |
| dx27 | atypical degenerated group of cells |
| dx27 | atypical epithelioid cells |
| dx27 | Atypical epithelial cells |
| dx27 | Atypical alveolar epithelial cells |
| dx27 | atypical glands |
| dx27 | atypical cell |
| dx27 | atypical single cells |
| dx27 | Atypical small cells |
| dx27 | atypical small/medium epithelial cells |
| dx27 | Atypical infiltrate |
| dx27 | Atypical glandular epithelium |
| dx27 | Atypical glandular cells |
| dx27 | Atypical respiratory cells |
| dx27 | Atypical bronchial cells |
| dx27 | Atypical squamous cell |
| dx27 | atypical keratinized squamous cells |
| dx27 | Atypical squamous epithelial |
| dx27 | Atypical squamous epithelium |
| dx27 | atypical squamoid cells |
| dx27 | atypical squamous metaplastic cells |
| dx27 | atypical bronchioalveolar lining cells |
| dx27 | atypical group of cells |
| dx27 | atypical large epithelial cells |
| dx27 | atypical scanty material with cells |
| dx27 | minimal cytologic atypia |
| dx27 | abnormal cells |
| dx27 | suspicious for a neoplasm |
| dx27 | suspicious for malignancy |
| dx27 | suspect for malignancy |
| dx27 | suggestive of malignancy |
| dx27 | cannot rule out malignancy |
| dx27 | Cannot exclude malignancy |
| dx27 | malignancy cannot be excluded |
| dx27 | cannot confidently exclude malignancy |
| dx27 | malignancy not excluded |
| dx28 | Suspicious for a Non-Hodgkin's lymphoma |
| dx28 | Suspicious for non-small cell carcinoma |
| dx28 | Suspicious for non small cell carcinoma |
| dx28 | Suspicious for nonsmall cell carcinoma |
| dx28 | Suspicious for poorly differentiated carcinoma |
| dx28 | Suspicious for small cell carcinoma |
| dx28 | Suspicious for adenocarcinoma |
| dx28 | Suspicious for squamous cell carcinoma |
| dx28 | Suspicious for squamous carcinoma |
| dx28 | Suspicious for lymphoma |
| dx28 | Suspicious for carcinoma |
| dx29 | metastatic |
| dx29 | metastasis |
| dx40 | thymoma |
| dx41 | sclerosing hemangioma |
| dx41 | pneumocytoma |
| dx50 | carcinoma |
| dx60 | necrosis |
| dx60 | necrotic |
| dx60 | necrotizing inflammation |
| dx65 | granulation tissue |
| dx66 | squamous papilloma |
| dx67 | vegetable matter |
| dx70 | Benign. |
| dx70 | Benign Cellular aspirate |
| dx70 | Benign Moderately cellular aspirate |
| dx70 | favour benign |
| dx70 | favor benign |
| dx70 | favour reactive |
| dx70 | favor reactive |
| dx70 | Benign lymph node |
| dx70 | Benign anthracotic lymph node |
| dx70 | Benign, scanty aspirate |
| dx70 | Scantly cellular aspirate consisting of occasional lymphocytes |
| dx70 | Reactive ciliated respiratory and reserve cells present. |
| dx70 | Benign-appearing bronchial tissue |
| dx70 | Benign bronchial tissue |
| dx70 | unremarkable bronchial mucosa |
| dx70 | Benign bronchial mucosa |
| dx70 | benign bronchial epithelium |
| dx70 | Mainly bronchial cells present |
| dx70 | Bronchial mucosa with no pathologic finding |
| dx70 | Benign-appearing respiratory mucosa |
| dx70 | Benign respiratory mucosa |
| dx70 | unremarkable endobronchial tissue |
| dx70 | Unremarkable endobronchial mucosa |
| dx70 | Unremarkable bronchial wall tissue |
| dx70 | Unremarkable tiny fragments of endobronchial mucosa |
| dx70 | BENIGN RESPIRATORY EPITHELIUM |
| dx70 | respiratory epithelium without significant diagnostic abnormality |
| dx70 | Unremarkable fragments of cartilage and endobronchial mucosa |
| dx70 | UNREMARKABLE CARTILAGE AND BRONCHIAL TISSUE |
| dx70 | Benign bronchial wall |
| dx70 | Bronchial mucosa without findings |
| dx70 | Benign chronic inflamed respiratory mucosa |
| dx70 | Benign, chronic inflamed bronchial mucosa |
| dx70 | Benign edematous bronchial tissue |
| dx70 | Edematous bronchial mucosa with no other specific pathology |
| dx70 | benign pulmonary parenchyma |
| dx70 | unremarkable lung parenchyma |
| dx70 | Benign lung tissue |
| dx70 | Benign alveolar lung tissue |
| dx70 | Benign alveolar lung parenchyma |
| dx70 | Benign-appearing lung parenchyma |
| dx70 | Benign lung parenchyma |
| dx70 | Benign parenchymal lung tissue |
| dx70 | Lung parenchymal tissue with no significant histological abnormality |
| dx70 | Bronchial and lung alveolar tissue without specific diagnostic abnormality |
| dx70 | Benign bronchial and alveolar tissue |
| dx70 | Benign bronchial and alveolar lung tissue |
| dx70 | Benign parenchymal lung |
| dx70 | benign lung tissue |
| dx70 | Benign lung and bronchial parenchyma |
| dx70 | Alveolar tissue with no significant findings |
| dx70 | Alveolar lung tissue without diagnostic abnormality |
| dx70 | very small amount of bland epithelium |
| dx70 | BENIGN FIBROADIPOSE TISSUE AND BLOOD |
| dx70 | Alveolar tissue without significant findings |
| dx70 | Mild chronic inflammation, no specific findings |
| dx70 | Minimal chronic inflammation, no other findings |
| dx70 | Reactive bronchial cells |
| dx70 | bronchial cells present in a background of lymphocytes |
| dx70 | lymphoid material with rare anthracotic-laden macrophages |
| dx70 | benign ciliated respiratory cells only |
| dx71 | Organized pneumonia |
| dx71 | Organizing pneumonia |
| dx71 | Bronchopneumonia with focal organization |
| dx72 | granuloma |
| dx72 | GRANULOMMATOUS INFLAMMATION |
| dx72 | epithelioid histiocytes |
| dx72 | suggestive of granulommatous process |
| dx72 | likely a granulommatous process |
| dx73 | Nonspecific scar |
| dx73 | suggestive of scar |
| dx73 | Scarred lung tissue |
| dx73 | Scarred alveolar lung |
| dx73 | scarlike tissue |
| dx73 | Scarring |
| dx73 | Scarred lung |
| dx73 | scar tissue |
| dx73 | Dense scar |
| dx73 | Fibroelastotic scar |
| dx73 | Bland fibrosis |
| dx74 | Hamartoma |
| dx75 | solitary (isolate) fibrous tumour |
| dx75 | solitary fibrous tumour |
| dx75 | solitary fibrous tumor |
| dx75 | solitary/localized fibrous tumor |
| dx76 | pleural plaque |
| dx77 | Langerhans Cell Histiocytosis |
| dx78 | Amyloid |
| dx79 | Mixed population of lymphocytes |
| dx79 | Benign lymphocytes |
| dx79 | Mainly lymphocytes noted |
| dx79 | Benign, consistent with lymph node |
| dx79 | benign mixed lymphocytes |
| dx79 | benign aspirate consisting of occasional lymphocytes |
| dx79 | Normal lymph node elements identified |
| dx79 | benign lymphoid material present |
| dx79 | benign lymph nodal tissue |
| dx79 | Only scant lymphocytes noted |
| dx79 | Benign, reactive lymphoid proliferation |
| dx79 | Lymphocytic infiltrate, favor reactive or inflammatory process |
| dx79 | Lymphocytic infiltrate, favour benign |
| dx79 | Bronchial mucosa showing mild acute and chronic inflammation |
| dx79 | Bronchial mucosa with mild chronic inflammation and some cartilage |
| dx79 | Bronchial mucosa with mild chronic inflammation |
| dx79 | Endobronchial mucosa with mild chronic inflammation |
| dx79 | lung alveolar tissue showing mild chronic inflammation |
| dx79 | Bronchial tissue showing chronic inflammation |
| dx79 | Bronchial tissue showing mild chronic inflammation |
| dx79 | Respiratory mucosa with mild chronic inflammation |
| dx79 | Lung parenchyma with fragment of cartilage and calcification |
| dx79 | Benign-appearing fibroadipose tissue |
| dx79 | Bronchial mucosa with eosinophilia |
| dx79 | Mainly blood with lymphocytes |
| dx79 | lymphocytes intermixed with bronchial cells |
| dx79 | mainly benign lymphoid material |
| dx79 | Mainly lymphocytes are present |
| dx79 | consists of lymphoid material |
| dx79 | Bronchial cells present in a background of lymphocytes |
| dx79 | Benign appearing lymphocytes |
| dx79 | Polymorphous lymphocytes |
| dx79 | mixed population of few lymphocytes present |
| dx79 | Scant lymphocytes present |
| dx79 | scant mixed lymphocytes noted |
| dx80 | No diagnostic abnormality |
| dx80 | No significant pathology |
| dx80 | no specific pathologic diagnosis |
| dx80 | no specific pathological diagnosis |
| dx80 | NO DEFINITE PATHOLOGICAL DIAGNOSIS |
| dx80 | no histopathological abnormality |
| dx80 | no significant abnormality |
| dx80 | No significant findings |
| dx80 | No findings |
| dx80 | no significant pathological abnormality |
| dx80 | No Significant Pathological Changes |
| dx80 | no significant histological abnormality |
| dx80 | No pathologic diagnosis |
| dx80 | no apparent lesional tissue |
| dx80 | no lesional tissue |
| dx80 | without significant pathology |
| dx80 | negative for significant pathology |
| dx80 | without significant histopathologic abnormality |
| dx87 | thyroid transcription factor |
| dx88 | melanoma markers |
| dx88 | melanoma cocktail |
| dx90 | Negative for malignancy |
| dx90 | NEGATIVE for evidence of malignancy |
| dx90 | Negative for malignant cells |
| dx90 | No tumor or malignancy |
| dx90 | No obvious malignant cells |
| dx90 | No definitive malignant cells |
| dx90 | No overtly malignant cells |
| dx90 | No clearly malignant cells |
| dx90 | No viable malignant cells |
| dx90 | No obviously malignant cells |
| dx90 | No malignant cells |
| dx90 | No evidence of definite malignancy |
| dx90 | No malignancy identified |
| dx90 | No malignancy in these biopsies |
| dx90 | No malignancy is seen |
| dx90 | NO EVIDENCE OF MALIGNANCY |
| dx90 | No evidence of dysplasia and malignancy |
| dx90 | No definite evidence of malignancy |
| dx90 | No malignancy is identified |
| dx90 | No evidence of dysplasia or malignancy |
| dx90 | Negative for dysplasia or malignancy |
| dx90 | Negative for dysplasia and malignancy |
| dx90 | no evidence of inflammation, dysplasia or malignancy |
| dx90 | Negative for epithelial malignancy |
| dx90 | Negative for carcinoma |
| dx91 | no obvious epithelial neoplasm identified |
| dx91 | negative for lung tissue or neoplasm |
| dx91 | negative for neoplasia |
| dx91 | No evidence of neoplasia |
| dx91 | no evidence of a tumour |
| dx91 | No tumor present |
| dx91 | No tumor identified |
| dx91 | No tumors identified |
| dx91 | Negative for neoplasm |
| dx91 | No neoplastic tissue |
| dx91 | No evidence of viable neoplasia |
| dx91 | Negative for primary or metastatic malignancy |
| dx92 | No evidence of metastatic malignancy |
| dx92 | No evidence of metastatic disease |
| dx92 | Negative for metastatic malignancy |
| dx92 | No metastatic disease |
| dx92 | no evidence of metastatic malignancy |
| dx92 | no evidence of metastasis |
| dx92 | Negative for metastasis |
| dx92 | Negative for metastatic carcinoma |
| dx93 | No evidence of metastatic or granulomatous disease |
| dx93 | No evidence of granulomatous disease or metastatic malignancy |
| dx93 | Negative for granulomatous disease or metastatic malignancy |
| dx93 | Negative for metastatic malignancy or granulomatous disease |
| dx94 | negative for lymphoma |
| dx94 | negative for lymphoma or neoplasia |
| dx95 | likely not representative |
| dx95 | may not be representative |
| dx95 | may not be a representative tissue |
| dx95 | not representative of the lesion |
| dx95 | Clinical correlation required regarding representation |
| dx95 | ? representative |
| dx95 | not a representative sample |
| dx95 | Suboptimal specimen |
| dx95 | Suboptimal for cytological assessment |
| dx95 | Sub-optimal for cytologic interpretation |
| dx99 | Insufficient lymphoid material |
| dx99 | Insufficient lymphoid tissue for cytological interpretation |
| dx99 | Insufficient lymphoid material for cytological interpretation |
| dx99 | Insufficient nodal tissue present |
| dx99 | Insufficient for evaluation |
| dx99 | Inadequate for cytological interpretation |
| dx99 | Insufficient for cytological interpretation |
| dx99 | Unsatisfactory material for cytological interpretation |
| dx99 | Unsatisfactory for proper evaluation |
| dx99 | Unsatisfactory due to insufficient cellularity |
| dx99 | Insufficient due to inadequate cellularity |
| dx99 | Insufficient due to scant cellularity |
| dx99 | Insufficient due to low cellularity |
| dx99 | Insufficient cellular material for proper assessment |
| dx99 | Specimen is insufficient for assessment |
| dx99 | Unsatisfactory because of inadequate cellularity |
| dx99 | Unsatisfactory for examination |
| dx99 | Inadequate material for cytological interpretation |
| dx99 | may not be a representative sample |
| dx99 | unsatisfactory sample |
| dx99 | Insufficient specimen for cytological evaluation |
| dx99 | Insufficient for cytological evaluation |
| dx99 | Unsatisfactory for cytological evaluation |
| dx99 | May not be a representative sample |
| dx99 | not specific |
| dx99 | no specific diagnosis |
| dx99 | Non-diagnostic |
| dx99 | Non diagnostic |
| dx99 | not diagnostic |
| dx99 | nondiagnostic |
| dx99 | Not a diagnostic specimen |
| dx99 | Unsatisfactory. |
| dx99 | Unsatisfactory specimen |
| dx99 | Unsatisfactory for cytological assessment |
| dx99 | Unsatisfactory for cytological interpretation |
| dx99 | Unsatisfactory for cytologic assessment |
| dx99 | Unsatisfactory due to scant cellularity |
| dx99 | not further diagnostic |
| dx99 | MARKEDLY DEGENERATE SAMPLE |
| dx99 | Material not assessable |
| dx99 | Insufficient tissue for pathologic evaluation |
| dx99 | No diagnostic material |
| dx99 | no diagnostic lung tissue |
| dx99 | No viable kidney tissue present |
| dx99 | crushed urothelial tissue |
| dx99 | Scanty crushed tissue |
| dx99 | insufficient for diagnosis |
| dx99 | Insufficient tissue for diagnosis |
| dx99 | Material insufficient |
| dx99 | INSUFFICIENT TISSUE FOR ASSESSMENT |
| dx99 | insufficient material |
| dx99 | Not a sufficient sample |
| dx99 | Inconclusive |
| dx99 | Tissue insufficient |
| dx99 | Tissue did not survive the processing |
| dx99 | No Tissue Material |
| dx99 | No tissue in the specimen |
| dx99 | No tissue is seen at microscopy |
| dx99 | Repeat biopsy may be of value |
| dx99 | Material insufficient for assessment |
| dx220 | from unknown primary |
| dx230 | pulmonary |
| dx230 | lung |
| dx231 | colon |
| dx231 | colonic |
| dx231 | cecum |
| dx231 | rectal |
| dx231 | colorectal |
| dx232 | breast |
| dx233 | urothelial carcinoma |
| dx233 | urothelial cell carcinoma |
| dx233 | transitional cell carcinoma |
| dx233 | bladder |
| dx234 | prostatic |
| dx234 | prostate |
| dx235 | thyroid |
| dx236 | melanoma |
| dx237 | kidney |
| dx237 | renal |
| dx238 | endometrial |
| dx238 | endometrium |
| dx238 | uterus |
| dx238 | uterine |
| dx239 | gallbladder |
| dx239 | biliary |
| dx240 | stomach |
| dx240 | gastric |
| dx241 | esophagus |
| dx241 | esophageal |
| dx242 | skin |
| dx243 | oral |
| dx243 | larynx |
| dx243 | laryngeal |
| dx243 | tonsilar |
| dx244 | ovarian |
| dx244 | ovary |
| dx245 | small intestinal |
| dx245 | small intestine |
| dx245 | small bowel |
| dx246 | pancreas |
| dx246 | pancreatic |
| dx247 | genitourinary |
| dx248 | GI origin |
| dx248 | gastrointestinal origin |
| dx248 | gastrointestinal tract |
| dx248 | foregut |
| dx249 | cervix |
| dx249 | cervical |
| dx250 | yolk sac tumour |
| dx250 | yolk sac tumor |
| dx250 | endodermal sinus tumour |
| dx250 | endodermal sinus tumor |
| dx251 | embryonal carcinoma |
| dx252 | germ cell tumour |
| dx252 | germ cell tumor |

Hierarchy of Diagnoses

| Supercedes | Subcedes | Overlap code | Description |
| --- | --- | --- | --- |
| dx5 | dx4 | 0 | non-small cell ca OVER small cell carcinoma |
| dx2 | dx5 | 0 | adenoca OVER NSCLC |
| dx3 | dx5 | 0 | SCC OVER NSCLC |
| dx2 | dx50 | 0 | adenoca OVER carcinoma |
| dx3 | dx50 | 0 | SCC OVER carcinoma |
| dx4 | dx50 | 0 | SmCC OVER carcinoma |
| dx5 | dx50 | 0 | NSmCC OVER carcinoma |
| dx7 | dx8 | 0 | atypical carcinoid OVER typical carcinoid |
| dx20 | dx28 | 0 | malignant OVER suspicous for X |
| dx90 | dx20 | 0 | neg for malignant cells OVER malignant cells |
| dx2 | dx20 | 0 | adenoca OVER malignant |
| dx3 | dx20 | 0 | SCC OVER mslignant |
| dx4 | dx20 | 0 | SmCC OVER malignant |
| dx5 | dx20 | 0 | NSmCC OVER malignant |
| dx70 | dx27 | 0 | benign/favour reactive OVER atypical |
| dx92 | dx30 | 0 | neg for met OVER met |
| dx93 | dx30 | 0 | neg for met+granuloma OVER met |
| dx93 | dx72 | 0 | neg for met+granuloma OVER granuloma |
| dx90 | dx50 | 0 | neg for malignancy/carcinoma OVER carcinoma NOS |
| dx28 | dx22 | 0 | susp for lymphoma OVER lymphoma |
| dx28 | dx2 | 0 | susp for adenoca OVER adenoca |
| dx28 | dx3 | 0 | susp for SCC OVER SCC |
| dx28 | dx5 | 0 | susp for NSCC OVER NSCC |
| dx28 | dx4 | 0 | susp for SmCC OVER SmCC |
| dx28 | dx50 | 0 | susp for carcinoma OVER carcinoma NOS |
| dx28 | dx27 | 0 | susp for X OVER atypical cells/less specific atypical |
| dx81 | dx72 | 0 | no granuloma OVER granuloma |
| dx82 | dx40 | 0 | neg for thymoma OVER thymoma |
| dx94 | dx22 | 0 | neg for lymphoma OVER lymphoma |
| dx87 | dx235 | -1 | thyroid transcription factor OVER thyroid |
| dx88 | dx236 | -1 | melanoma markers/melanoma cocktail OVER melanoma |
| ndx16 | ndx15 | 0 | anaplastic hemangiopericytoma OVER hemangiopericytoma |
| ndx24 | ndx25 | 0 | subependymoma OVER ependymoma |
| ndx06 | ndx08 | 0 | anaplastic ganglioglioma OVER ganglioglioma |
| ndx06 | ndx30 | 0 | anaplastic ganglioglioma OVER glioma |
| ndx08 | ndx30 | 0 | ganglioglioma OVER glioma |
| ndx26 | ndx03 | 0 | pilocytic astrocytoma OVER astrocytoma |
| ndx02 | ndx03 | 0 | anaplastic astrocytoma OVER astrocytoma |
| ndx04 | ndx05 | 0 | anaplastic oligodendroglioma OVER oligodendroglioma |
| ndx04 | ndx30 | 0 | anaplastic oligodendroglioma OVER glioma |
| ndx05 | ndx30 | 0 | oligodendroglioma OVER glioma |
| ndx10 | dx23 | 0 | gliosarcoma OVER sarcoma |
| ndx19 | ndx03 | 0 | pleomorphic xanthoastrocytoma OVER astrocytoma |
| ndx20 | ndx03 | 0 | xanthoastrocytoma OVER astrocytoma |
| ndx22 | ndx03 | 0 | oligoastrocytoma OVER astrocytoma |
| ndx19 | ndx20 | 0 | pleomorphic xanthoastrocytoma OVER xanthoastrocytoma |

Location Dictionary

| Location Category | Description |
| --- | --- |
| nlocrt | right |
| nloclf | left |
| nloc01 | frontal |
| nloc02 | parietal |
| nloc03 | temporal |
| nloc04 | occipital |
| nloc05 | cerebellum |
| nloc05 | cerebellar |
| nloc06 | fronto-temporal |
| nloc07 | fronto-parietal |
| nloc08 | temporo-parietal |
| nloc09 | fronto-ethmoid |
| nloc10 | suprasellar |
| nloc11 | sella |
| nloc12 | pituitary |
| nloc12 | pitiutary |
| nloc13 | CEREBELLOPONTINE ANGLE |
| nloc13 | CAP |
| nloc13 | CP angle |
| nloc14 | dura |
| nloc15 | skull - base |
| nloc15 | skull base |
| nloc16 | posterior fossa |
| nloc17 | medulla |
| nloc18 | pons |
| nloc19 | pineal |
| nloc20 | corpus callosum |
| nloc21 | clivus |
| nloc22 | nasopharyngeal mucosa |
| nloc23 | brainstem |
| nloc30 | ventricle |
| nloc30 | ventricular |
| nloc33 | 3rd ventricle |
| nloc33 | third ventricle |
| nloc34 | 4th ventricle |
| nloc34 | fourth ventricle |
| nloc35 | intraventricular |
| nloc38 | foramen |
| nloc40 | MCA |
| nloc40 | middle cerebral artery |
| nloc40 | M1 |
| nloc40 | M2 |
| nloc41 | ICA |
| nloc41 | internal carotid artery |
| nloc42 | basilar artery |
| nloc42 | basilar, clot |
| nloc42 | basilar clot |
| nloc99 | not otherwise specified |

### Appendix B

Mutual Exclusive Diagnostic Groups

| Brain tumour | ndx01, ndx02, ndx03, ndx04, ndx05, ndx06, ndx07, ndx08, ndx09, ndx10, ndx11, ndx12, ndx13, ndx14, ndx15, ndx16, ndx17, ndx18, ndx19, ndx20, ndx22, ndx23, ndx24, ndx25, ndx26, ndx27, ndx28, ndx30, ndx31, ndx32, ndx33, ndx34, ndx35, ndx36, ndx37, ndx38, ndx39, ndx40, ndx41, ndx42, ndx43, ndx44, ndx70, ndx71, ndx72, ndx73, ndx90 |
| --- | --- |
| Metastasis | dx02, dx03, dx04, dx05, dx06, dx07, dx10, dx20, dx21, dx22, dx23, dx25, dx28, dx29, dx50, dx77, dx220, dx230, dx231, dx232, dx233, dx234, dx235, dx236, dx237, dx238, dx239, dx240, dx241, dx242, dx243, dx244, dx245, dx246, dx247, dx248, dx249, dx250, dx251, dx252 |
| Other brain lesion | ndx29, ndx46, ndx47, ndx48, ndx49, ndx50, ndx52, ndx53, ndx54, ndx55, ndx56, ndx57, ndx58, ndx59, ndx60, ndx61, ndx65, ndx66, ndx67, ndx68, ndx80, ndx83, ndx84, ndx180, ndx181, ndx182, ndx190 |
| Brain cardiovascular disease | ndx81, ndx82, ndx130, ndx141, ndx142, ndx143, ndx144, ndx145, ndx146, ndx147, ndx148, ndx149, ndx150, ndx175, ndx176, ndx177, ndx178, ndx179 |
| Miscellaneous | ndx69, dx08, dx09, dx15, dx27, dx40, dx41, dx60, dx65, dx66, dx67, dx70, dx71, dx72, dx73, dx74, dx75, dx76, dx78, dx79, dx80, dx90, dx91, dx92, dx93, dx94, dx95, dx99 |
